# Efficient production of (*S*)-1-phenyl-1,2-ethanediol using xylan as co-substrate by a coupled multi-enzyme *Escherichia coli* system

**DOI:** 10.1186/s12934-020-01344-x

**Published:** 2020-04-07

**Authors:** Junchao Rao, Rongzhen Zhang, Guanyu Xu, Lihong Li, Yan Xu

**Affiliations:** 1 Key Laboratory of Industrial Biotechnology of Ministry of Education & School of Biotechnology, Jiangnan University, Wuxi, 214122 People’s Republic of China; 2 Xuteli School, Beijing Institute of Technology, Beijing, 100081 People’s Republic of China; 3 Present Address: School of Biotechnology, Jiangnan University, 1800 Lihu Avenue, Wuxi, 214122 China

**Keywords:** (*S*)-carbonyl reductase II, Glucose dehydrogenase mutant Ala258Phe, Endo-β-1,4-xylanase 2, Multi-enzyme system, Cofactor regeneration, Chiral synthesis

## Abstract

**Background:**

(*S*)-1-phenyl-1,2-ethanediol is an important chiral intermediate in the synthesis of liquid crystals and chiral biphosphines. (*S*)-carbonyl reductase II from *Candida parapsilosis* catalyzes the conversion of 2-hydroxyacetophenone to (*S*)-1-phenyl-1,2-ethanediol with NADPH as a cofactor. Glucose dehydrogenase with a Ala258Phe mutation is able to catalyze the oxidation of xylose with concomitant reduction of NADP^+^ to NADPH, while endo-β-1,4-xylanase 2 catalyzes the conversion of xylan to xylose. In the present work, the Ala258Phe glucose dehydrogenase mutant and endo-β-1,4-xylanase 2 were introduced into the (*S*)-carbonyl reductase II-mediated chiral pathway to strengthen cofactor regeneration by using xylan as a naturally abundant co-substrate.

**Results:**

We constructed several coupled multi-enzyme systems by introducing (*S*)-carbonyl reductase II, the A258F glucose dehydrogenase mutant and endo-β-1,4-xylanase 2 into *Escherichia coli*. Different strains were produced by altering the location of the encoding genes on the plasmid. Only recombinant *E. coli*/pET-G-S-2 expressed all three enzymes, and this strain produced (*S*)-1-phenyl-1,2-ethanediol from 2-hydroxyacetophenone as a substrate and xylan as a co-substrate. The optical purity was 100% and the yield was 98.3% (6 g/L 2-HAP) under optimal conditions of 35 °C, pH 6.5 and a 2:1 substrate-co-substrate ratio. The introduction of A258F glucose dehydrogenase and endo-β-1,4-xylanase 2 into the (*S*)-carbonyl reductase II-mediated chiral pathway caused a 54.6% increase in yield, and simultaneously reduced the reaction time from 48 to 28 h.

**Conclusions:**

This study demonstrates efficient chiral synthesis using a pentose as a co-substrate to enhance cofactor regeneration. This provides a new approach for enantiomeric catalysis through the inclusion of naturally abundant materials.

## Background

Optically active alcohols are versatile chiral compounds, widely utilized as intermediates in the fine chemical and pharmaceutical industries [1–3]. Alcohol dehydrogenase-mediated asymmetric reduction of ketones is the most efficient method for synthesis of chiral alcohols, with a theoretical yield of 100% [4, 5]. As an example, NADPH-dependent (*S*)-carbonyl reductase II (SCRII) from *Candida parapsilosis* CCTCC M203011 catalyzes the reduction of 2-hydroxyacetophenone (2-HAP) to (*S*)-1-phenyl-1,2-ethanediol (PED), an important chiral compound which can undergo stereoselective polymerization to form liquid crystals and chiral biphosphines [6, 7]. Cui et al. found that two new carbonyl reductases, 2,3-butanediol dehydrogenase from *Bacillus subtilis* and polyol dehydrogenase from *Gluconobacter oxydans* could convert 2-hydroxyacetophenon (2-HAP) to (*R*)-1-phenyl-1,2-ethanediol ((*R*)-PED) and (*S*)-1-phenyl-1,2-ethanediol ((*S*)-PED) with excellent stereochemical selectivity and strong substrate tolerance, respectively [8].

The reaction that is catalyzed by alcohol dehydrogenase is limited by cofactor recycling. To enhance the regeneration of cofactors, substrate-coupled and enzyme-coupled techniques are always included in chiral synthesis reactions, through the use of multi-enzyme systems [9, 10]. For example, Xu et al. described the preparation of ethyl (*S*)-4-chloro-3-hydroxybutanoate via the coupling of carbonyl reductase with mannitol or sorbitol dehydrogenase [11]. Kosjek et al. carried out asymmetric synthesis of 4,4-dimethoxytetrahydro-2H-pyran-3-ol using a ketone reductase and in situ cofactor recycling by glucose dehydrogenase (GDH) [12]. Yamamoto et al. reported the efficient production of ethyl (*S*)-4-chloro-3-hydroxybutanoate with over 99% optical purity by co-expressing carbonyl reductase from *Kluyveromyces aestuarii* and formate dehydrogenase in *Escherichia coli* [13]. Zhang et al. increased the production efficiency of (*R*)-PED by employing an enzyme-coupling system containing SCRII and GDH [14]. These systems enabled scientists to improve the efficiency of the enantioselective reaction with glucose or formate as co-substrates. However, the use of xylan in chiral synthesis to enhance cofactor recycling would be more interesting since they are naturally abundant materials.

Lignocellulose is well known as the most widely distributed and abundant polysaccharide in nature, which has been recognized as an attractive feedstock for the production of fuels and industrially important metabolites [15, 16]. Lignocellulosic biomass is mainly composed of cellulose, hemicellulose, and lignin [17]. Pretreatment with alkaline, acidic, and/or enzymatic hydrolysis causes hemicellulose to decompose to pentoses, mainly xylan and lignin [18, 19]. Xylan can be broken down to xylose by endo-β-1,4-xylanases (XYN2), and the expression and characterization of XYN2 from *Trichoderma reesei* Rut C-30 h has been reported [20].

**Fig. 1:**
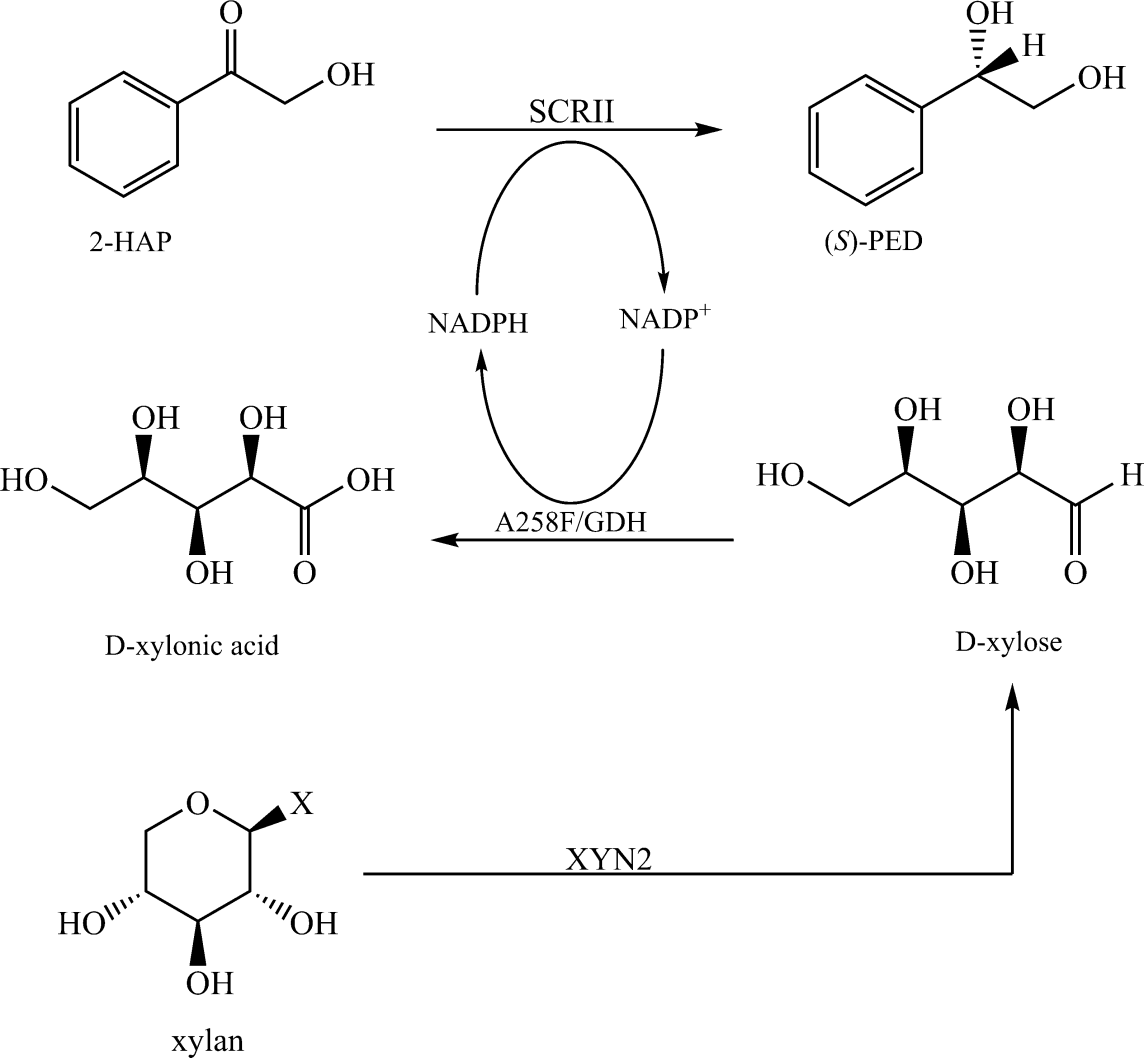
The biosynthetic pathway of (*S*)-PED through a multi-enzyme coupled system containing SCRII, A258F/GDH, and XYN2

We have previously isolated a GDH with good solvent-resistant from *Bacillus* sp. YX-1, and introduced this enzyme for cofactor regeneration in chiral synthesis with glucose as a co-substrate [21]. The Ala258Phe mutant of GDH exhibited altered cofactor preference and improved xylose binding ability while reducing NADP^+^ to NADPH [22]. An SCRII from *C. parapsilosis* has been shown to catalyze the conversion of 2-HAP to (*S*)-PED with NADPH as an electron donor [23]. The present study aimed to improve the efficiency of chiral synthesis by the construction of an NADPH-recycling multi-enzyme system containing SCRII, A258F/GDH and XYN2 from *T. reesei* Rut C-30 h (Fig. 1). XYN2 and A258F/GDH were introduced into the SCRII-mediated chiral synthesis pathway. In conditions of optimal pH, temperature and substrate-co-substrate ratio, the *E. coli*/pET-G-S-2 system performed well in terms of (*S*)-PED production without the addition of external cofactors. This work thus presents a novel strategy for cofactor recycling by introducing xylan into an enantioselective reaction pathway, which offers efficient chiral synthesis using an abundant natural chemical as a co-substrate.

## Results and discussion

### Construction of the multi-enzyme-coupled-system containing SCRII, A258F/GDH and XYN2

**Fig. 2:**
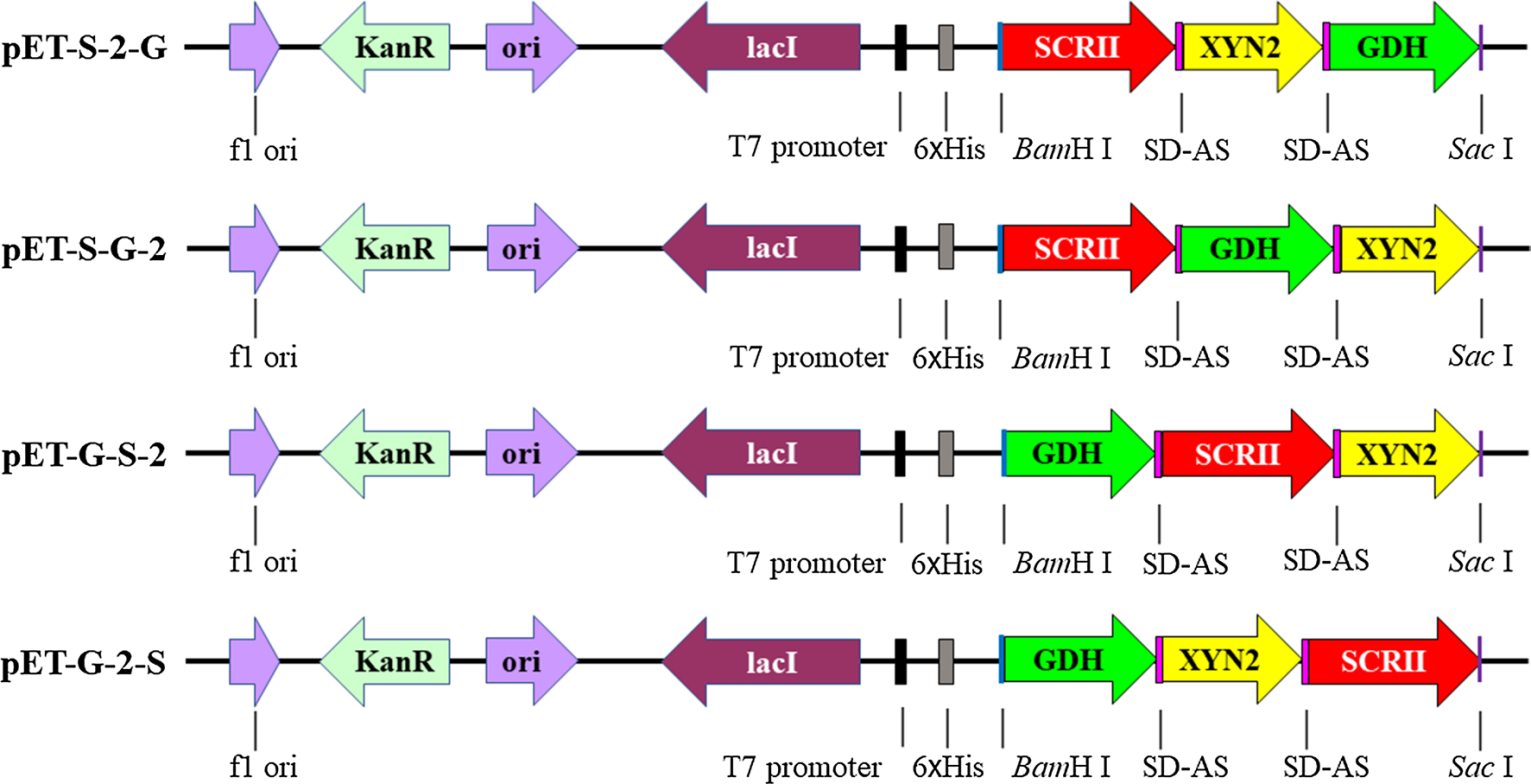
The location of SCRII, A258F/GDH, and XYN2 on plasmid pET-28a

We successfully constructed four recombinant plasmids pET-S-2-G, pET-S-G-2, pET-G-2-S and pET-G-S-2 (Fig. 2), in which the three genes were located in different sites of the pET-28a plasmid. For example, SCRII was located nearest to and A258F/GDH farthest from the promoter, while XYN2 was located between SCRII and A258F/GDH in pET-S-2-G. Transformation into *E. coli* BL21 (DE3) competent cells resulted in the generation of *E. coli*/pET-S-2-G, *E. coli*/pET-S-G-2, *E. coli*/pET-G-2-S and *E. coli*/pET-G-S-2 strains, which were verified by DNA sequencing.

### All three enzymes were successfully expressed in *E. coli*/pET-G-S-2 with no effect on cell growth

**Fig. 3:**
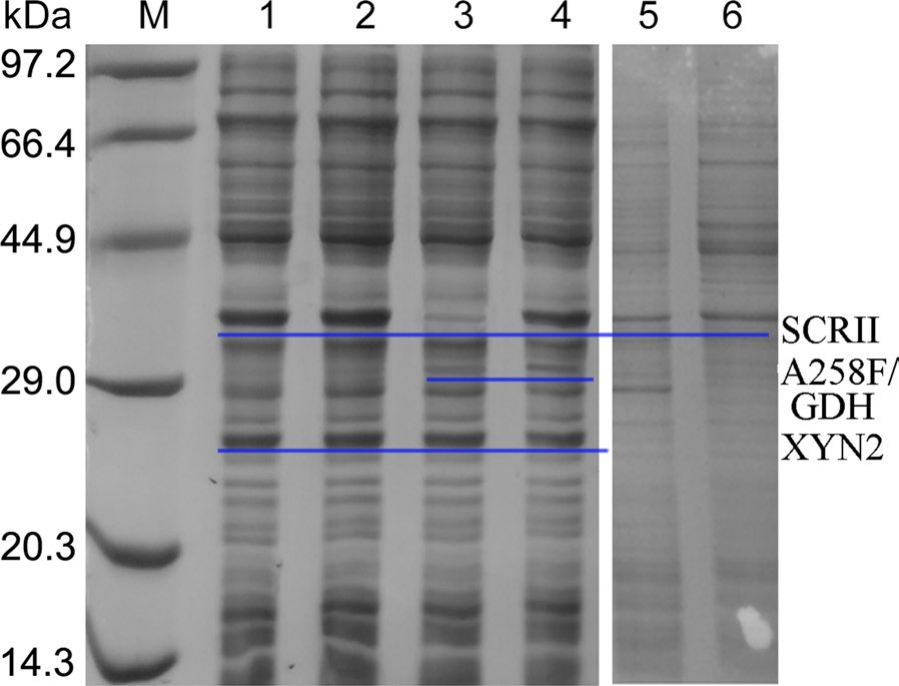
SDS-PAGE analysis of protein expression. M. Protein marker. The cell-free extracts of *E. coli*/pET-S-G-2 (Lane 1), *E. coli*/pET-S-2-G (Lane 2), *E. coli*/pET-G-2-S (Lane 3), *E. coli*/pET-G-S-2 (Lane 4), *E. coli*/pET-28a (Lane 5) induced with 0.1 mM IPTG; lane 6. *E. coli*/pET-G-S-2 without induction

Sodium dodecyl sulfate polyacrylamide gel electrophoresis (SDS-PAGE) analysis revealed that three predominant bands corresponding to the theoretical sizes (33, 30 and 22 kDa) of the target recombinant enzymes (SCRII, A258F/GDH and XYN2, respectively) were only present in the cell-free extracts of *E. coli*/pET-G-S-2. However, no expression of A258F/GDH was observed in *E. coli*/pET-S-2-G or *E. coli*/pET-S-G-2, and SCRII could not be expressed in *E. coli*/pET-G-2-S (Fig. 3). The A258F/GDH and SCRII enzymes were not able to be expressed in *E. coli*/pET-S-2-G, *E. coli*/pET-S-G-2 or *E. coli*/pET-G-2-S, which might be due to their location on the plasmid or their distance from the promoter. Kim et al. reported that the genes at the back of the promoter are expressed at lower levels than those at the front [24], indicating that protein expression levels are significantly influenced by the order of their coding genes on the plasmid. Moreover, the SD-AS sequence initiates translation, which might contribute to the differential expression of SCRII, A258F/GDH and XYN2 in *E. coli* [25].

**Fig. 4:**
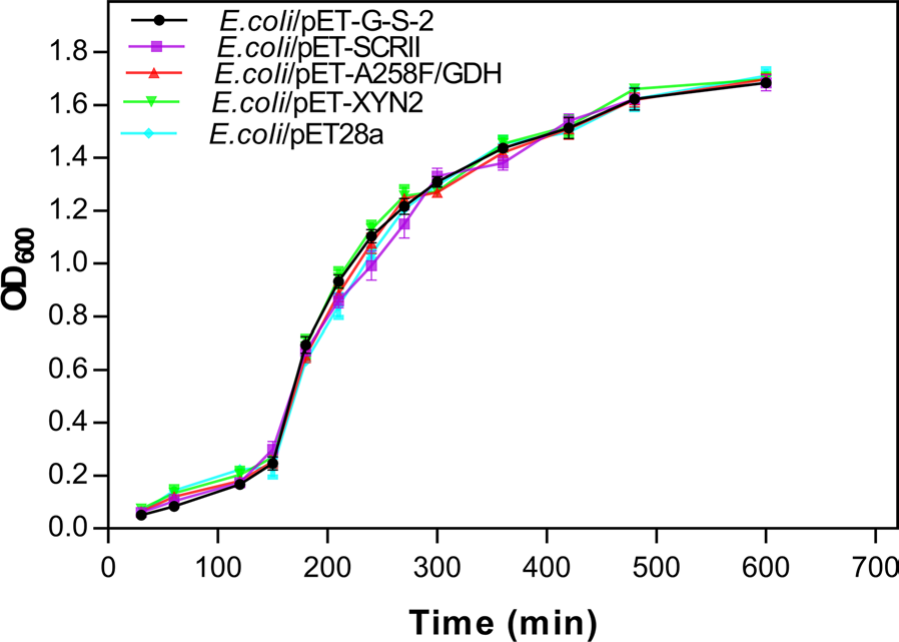
Cell-growth curve of the recombinant strains. The recombinant strains were cultured at 37 °C in 500 mL flask bottles, with an initial working volume of 150 mL, and then 0.1 mM IPTG was added at 3 h to induce protein expression. The induction temperature is 25 °C

Turbidity measurements revealed that *E. coli*/pET-G-S-2, *E. coli*/pET-SCRII, *E. coli*/pET-A258F/GDH, *E. coli*/pET-XYN2 and the control strain *E. coli*/pET-28a all had similar growth trends (Fig. 4). This suggests that the expression of A258F/GDH and XYN2 in *E. coli* does not affect cell growth. Therefore, we used *E. coli*/pET-G-S-2 expressing SCRII, A258F/GDH and XYN2 for further experiments and chiral synthesis.

### The functions of SCRII and A258F/GDH are well balanced

The activities of all three enzymes in the cell-free extracts of recombinant strains are summarized in Table 1. The activities of SCRII, A258F/GDH and XYN2 from *E. coli*/pET-G-S-2 were found to be 0.85, 1.36 and 55.33 U/mg toward 2-HAP, xylose and xylan, respectively. The activities toward 2-HAP, xylose and xylan of *E. coli*/pET-G-S-2, which expressed all three enzymes, was lower than that of *E. coli/*pET-SCRII, *E. coli/*pET-A258F/GDH and *E. coli*/pET-XYN2, which only expressed (Table 1). The activity of XYN2 from *E. coli*/pET-XYN2 toward xylan was much higher than that of SCRII from *E. coli*/pET-SCRII or A258F/GDH from *E. coli*/pET-A258F/GDH, although SCRII and A258F/GDH exhibited similar activity toward their corresponding substrates. These results suggest that the functions of SCRII for chiral catalysis and GDH for cofactor regeneration are balanced.

**Table 1:** Enzyme activities in the cell-free extracts of recombinant strains

Strains	Specific activities (U/mg)		
	Towards 2-HAP	Towards xylose	Towards xylan
*E. coli* BL21/pET-SCRII	2.73 ± 0.01	ND	ND
*E. coli* BL21/pET-A258F/GDH	ND	6.85 ± 0.02	ND
*E. coli* BL21/pET-XYN2	ND	ND	267.51 ± 11.8
*E. coli* BL21/pET-S-G-2	1.54 ± 0.01	ND	65.13 ± 2.2
*E. coli* BL21/pET-S-2-G	1.28 ± 0.02	ND	54.45 ± 3.1
*E. coli* BL21/pET-G-S-2	0.85 ± 0.02	1.36 ± 0.01	55.33 ± 4.3
*E. coli* BL21/pET-G-2-S	ND	1.18 ± 0.02	35.93 ± 2.6

*ND* not detected

Analysis of the activities of SCRII, A258F/GDH and XYN2 in *E. coli*/pET-G-S-2 were evaluated under different conditions, which revealed the optimum pH to be 6.5, 6.5 and 5.0 and the optimal temperature to be 35, 50 and 50 °C for SCRII, A258F/GDH and XYN2, respectively. Under the optimal conditions, the activities of SCRII, A258F/GDH and XYN2 in *E. coli*/pET-G-S-2 were 2.73, 6.85 and 267.51 U/mg.

The activities of all three enzymes could be detected only in *E. coli*/pET-G-S-2, which also confirmed that the three enzymes were all expressed only in *E. coli*/pET-G-S-2. This may be due to the enzyme activity of A258F/GDH was higher than that of SCRII when expressed alone, and the difference was caused by the different positions of SCRII and A258F/GDH on the vector. In the recombinant strain *E. coli*/pET-G-S-2, the enzyme activity of SCRII was similar to that of A258F/GDH, while the three enzymes could not be all expressed in the other recombinant strains. As a result, the function of target enzymes SCRII and A258F/GDH in recombinant *E. coli/*pET-S-G-2 was more balanced than that in the other three recombinant strains *E. coli*/pET-S-G-2, *E. coli*/pET-S-2-G and *E. coli*/pET-G-S-2 [14].

### Optimization of biotransformation of (*S*)-PED by *E. coli*/pET-G-S-2

Since the optimal temperature and pH conditions were different for each enzyme, the conditions for biotransformation of 2-HAP to (*S*)-PED by *E. coli*/pET-G-S-2 required further optimization. When the cell concentration was 10% (w/v), the yield of (*S*)-PED from 6 g/L 2-HAP reached 85.8% in 24 h using *E. coli*/pET-G-S-2, while the yield from *E. coli*/pET-SCRII was only 63.6% at 24 h [23].

**Fig. 5:**
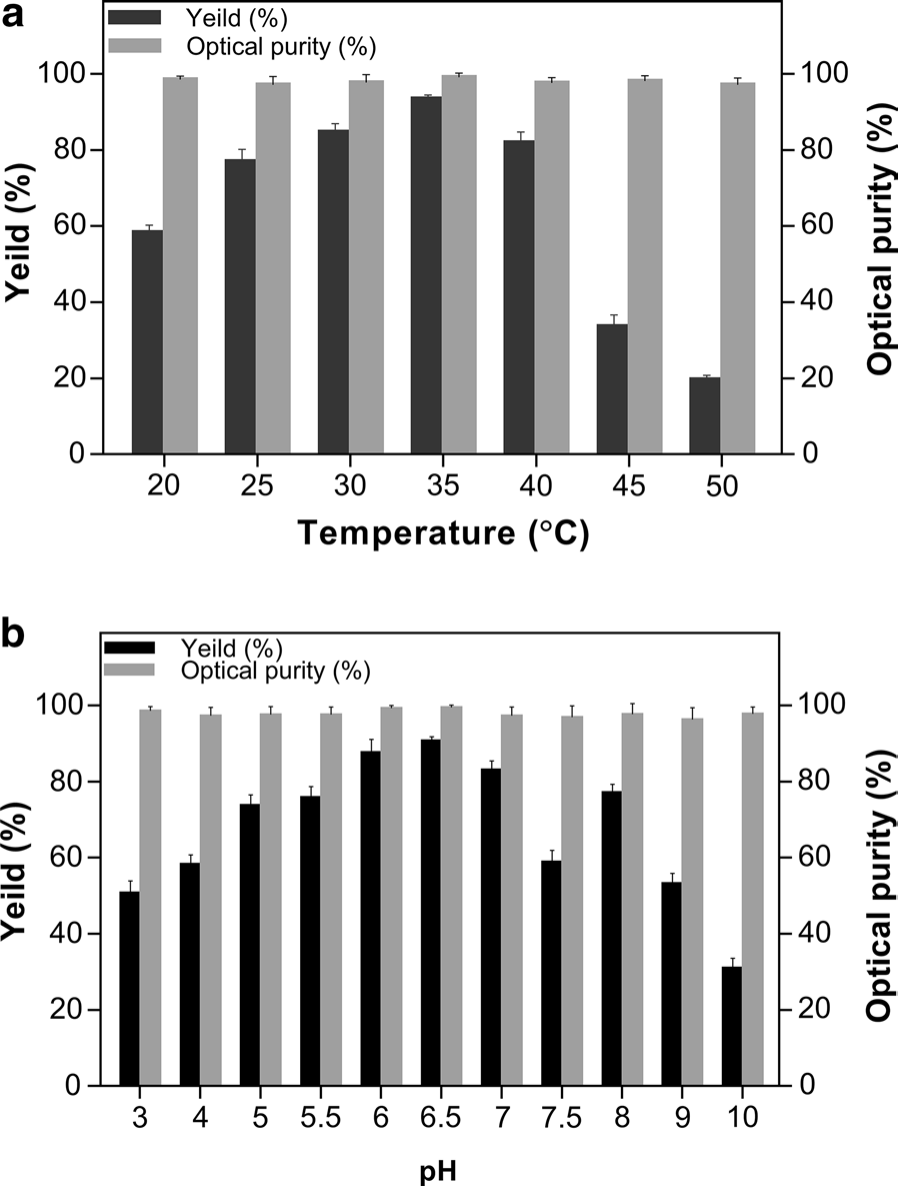
The effects of temperature (**a**) and pH (**b**) on *E. coli* BL21/pET-G-S-2 catalyzing 2-HAP biotransformation. The temperature optimum of 2-HAP transformation was determined at various temperatures (20–50 °C). The pH optimum of 2-HAP transformation was determined between pH 3.0 and 10.0 using 0.1 M citric acid buffer (pH 3.0, 4.0, 5.0, 6.0, 6.5), 0.1 M potassium phosphate buffer (pH 6.5, 7.0, 7.5), and 50 mM Tris–HCl buffer (pH 8.0, 9.0, 10.0)

Phillips et al. reported the significant effect of pH on the enantiospecificity of a secondary alcohol dehydrogenase, and demonstrated that the pH affects the enzyme–substrate binding strength [26]. As depicted in Fig. 5a, the present study revealed that the yield of (*S*)-PED from *E. coli*/pET-G-S-2 was increased with the increased pH value between 3.0 and 6.5. At the optimal pH of 6.5, biotransformation of (*S)*-PED was achieved with an optical purity of 100% and a yield of 90.5% when 6 g/L 2-HAP was used as substrate (Fig. 5a).

Temperature also has a significant influence on enzyme activity [27], and can affect product stereoselectivity during enzyme-catalyzed chiral biosynthesis [28]. As shown in Fig. 5b, we found that the optimal temperature for the biotransformation of (*S*)-PED by *E. coli*/pET-G-S-2 was 35 °C, at which the optical purity was 100% and the yield was 93.6% when the substrate 2-HAP was 6 g/L. The optimal temperature of *E. coli/*pET-SCRII has been reported to be 35 °C [23], while *E. coli/*pET-A258F/GDH exhibited the highest activity toward xylose at 55 °C [22] and the optimal temperature for the activity of XYN2/*E. coli* toward xylan has been shown to be 50 °C [20]. In the present study, we found the activity of SCRII to decrease rapidly with increasing temperature, while the activity of other two enzymes A258F/GDH and XYN2 remained active at 35 °C.

**Fig. 6:**
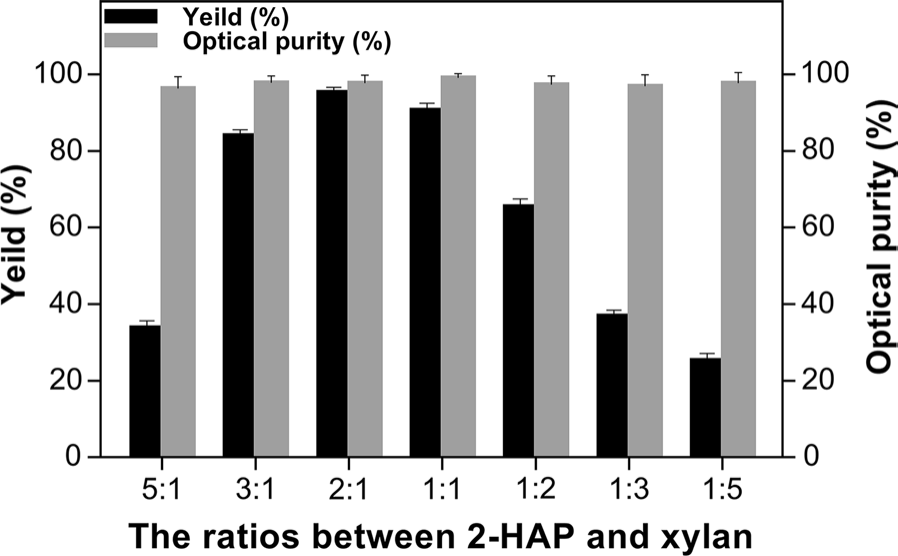
Optimization of the ratio between 2-HAP and xylan for (*S*)-PED biotransformation by the whole-cells of *E. coli*/pET-G-S-2

Cai et al. described the impact of different substrate-co-substrate ratios on the biotransformation efficiency of recombinant enzymes [29]. Although we found SCRII and A258F/GDH from *E. coli*/pET-G-S-2 to had similar activities (0.85 and 1.36 U/mg, respectively), XYN2 exhibited considerably higher activity of 55.33 U/mg. Therefore, we optimized the ratio of substrate (2-HAP) and co-substrate xylan. As illustrated in Fig. 6, when the ratio of 2-HAP and xylan was 2:1, *E. coli*/pET-G-S-2 catalyzed the biotransformation of (*S)*-PED with the highest optical purity (100%) and yield (95.8%) with 6 g/L 2-HAP as substrate in 24 h.

Both temperature and pH could affect the yield and optical purity of asymmetric reduction reactions [26–28], while SCRII was an enzyme with unusual stereospecificity catalyzing an anti-Prelog reduction of 2-HAP to (*S*)-PED [6]. Therefore, through the optimization of asymmetric reduction reaction conditions, the temperature and pH had a great influence on the yield and hardly affected the optical purity.

As a control, we also investigated the biosynthesis of (*S*)-PED by recombinant strains *E. coli*/pET-S-G-2, *E. coli*/pET-G-2-S and *E. coli*/pET-S-2-G under optimal reaction conditions. However, we did not detect the production of (*S*)-PED in 24 h by *E. coli*/pET-G-2-S. The recombinant *E. coli*/pET-S-G-2 and *E. coli*/pET-S-2-G synthesize (*S*)-PED in a slightly lower yield in 24 h than *E. coli*/pET-SCRII.

### Efficient transformation of (*S*)-PED by *E. coli*/pET-G-S-2

**Fig. 7:**
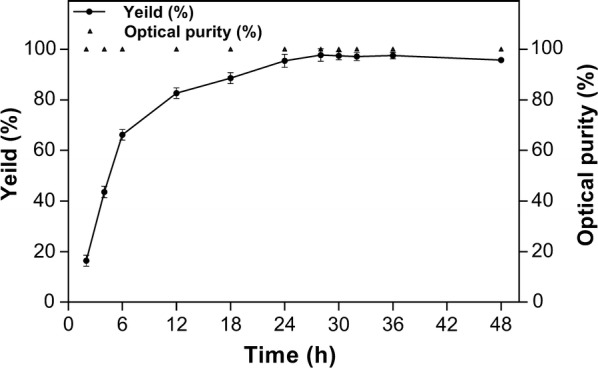
Time courses of biotransformation of 2-HAP to (*S*)-PED by the whole-cells of *E. coli*/pET-G-S-2. The enantioselective reaction was performed under optimal conditions: pH 6.5 and 35 °C

We investigated the reaction duration of the enzyme-coupled system *E. coli*/pET-G-S-2 by targeting the highest optical purity and yield of (*S*)-PED under optimal conditions of pH, temperature and substrate-co-substrate ratio. The yield of (*S*)-PED was highest (98.3%) with an optical purity of 100% at 28 h when the substrate 2-HAP was 6 g/L (Fig. 7). Compared with *E. coli*/pET-SCRII, the introduction of XYN2 and A258F/GDH into the SCRII-mediated chiral synthesis pathway in *E. coli*/pET-G-S-2 caused a 54.6% increase in yield and the time required for the reaction to complete was significantly reduced from 48 to 28 h [23]. The results of the present study suggest that the introduction of XYN2 and A258F/GDH accelerates the bioconversion of 2-HAP to (*S*)-PED in SCRII-mediated chiral synthesis. Efficiently catalysis of (*S*)-PED biosynthesis with absolute stereochemical selectivity (100% optical purity) was achieved using *E. coli*/pET-G-S-2. Furthermore, biotransformation of (*S*)-PED by *E. coli*/pET-G-S-2 was upscaled in 200 mL. It produced (*S*)-PED with an optical purity of > 99.9% and a yield of 96.8% when 6 g/L 2-HAP was used as substrate. Thus, the multi-enzyme-coupled system *E. coli*/pET-G-S-2 efficiently catalyzes the stereospecific reduction of 2-HAP to (*S*)-PED. Ye et al. reported the improved efficiency of ethyl (*S*)-4-chloro-3-hydroxybutanoate synthesis through the coupling of carbonyl reductase and GDH [30]. Kosjek et al. performed asymmetric synthesis of 4,4-dimethoxytetrahydro-2H-pyran-3-ol by co-expressing a ketone reductase and glucose dehydrogenase [12]. These approaches both achieved increased cofactor regeneration, which could overcome the limitation of coenzyme restriction for asymmetric synthesis and accelerate the initial reaction rate, resulting in improved catalytic efficiency. The present work describes a simple approach to achieve cofactor regeneration in chiral synthesis using a naturally abundant co-substrate.

## Conclusions

To improve the efficiency of chiral synthesis, A258F/GDH and XYN2-mediated cofactor regeneration were introduced into the SCRII-catalyzed chiral synthesis pathway. Several multi-enzyme-coupled systems containing SCRII, A258F/GDH and XYN2 were constructed by changing the gene locations within the plasmid. The three enzymes were all successfully expressed in the recombinant strain, *E. coli*/pET-G-S-2. The biotransformation of 2-HAP to (*S*)-PED was achieved with a high optical purity (100%) in a high yield (98.3%) with 6 g/L 2-HAP in 28 h. The introduction of XYN2 and A258F/GDH into the asymmetric reaction dramatically improved the efficiency of chiral biosynthesis. This work provides a foundation for future studies of efficient enantioselective synthesis using an abundant natural compound, xylan, as a co-substrate to enhance cofactor recycling.

## Materials and methods

### Microorganisms and chemicals

The PrimeSTAR^®^MAX, pMD19-T vector, restriction enzymes and T4 DNA ligase were bought from Takara (Shanghai, China). 2-HAP was purchased from TCI Development Co., Ltd. (Shanghai, China). (*R*)-, (*S*)-PED, PED and NADPH were purchased from Sigma-Aldrich (Shanghai, China). Hexane and isopropanol of chromatographic grade used for high performance liquid chromatography (HPLC) were purchased from Sigma-Aldrich (Shanghai, China). All other chemicals used were of analytical grade and commercially available.

*Escherichia coli* JM109 (Invitrogen Co., Shanghai, China) was used as a host for plasmid propagation. *E. coli* BL21 (DE3) (Invitrogen Co., Shanghai, China) was used for protein expression. *E. coli* JM109 and *E. coli* BL21 were cultured at 37 °C in Luria–Bertani (LB) medium supplemented with kanamycin (50 μg/mL) as the selective marker. The strains and plasmids used in this work were summarized in Table 2.

**Table 2:** Bacterial strains, plasmids and primers used in this work

Strains/plasmids/primers	Characteristics	Sources
Strains		
*E. coli* JM109	Host gene cloning	Invitrogen
*E. coli* BL21(DE3)	Host of target gene for expression	Invitrogen
*E. coli*/pET-A258F/GDH	*E. coli* BL21 containing pET-A258F/GDH	[22]
*E. coli*/pET-SCRII	*E. coli* BL21 containing pET-SCRII	[23]
*E. coli*/pET-XYN2	*E. coli* BL21 containing pET-XYN2	This work
*E. coli*/pET-S-G-2	*E. coli* BL21 containing pET-S-G-2	This work
*E. coli*/pET-S-2-G	*E. coli* BL21 containing pET-S-2-G	This work
*E. coli*/pET-G-S-2	*E. coli* BL21 containing pET-G-S-2	This work
*E. coli*/pET-G-2-S	*E. coli* BL21 containing pET-G-2-S	This work
Plasmids		
pET-A258F/GDH	A258F/GDH gene on pET-28a, 6.15 kb	This lab
pET-SCRII	SCRII gene on pET-28a, 6.21 kb	This lab
pET-XYN2	XYN2 gene on pET-28a, 6.04 kb	This work
pMD19-T	Cloning plasmid, 2.7 kb, Amp^r^	Takara Co.
T-S-G-2	S-G-2 gene on pMD19-T,4.97 kb	This work
T-S-2-G	S-2-G gene on pMD19-T, 4.97 kb	This work
T-G-S-2	G-S-2 gene on pMD19-T, 4.97 kb	This work
T-G-2-S	G-2-S gene on pMD19-T, 4.97 kb	This work
pET-S-G-2	S-G-2 gene on pET-28a 7.66 kb	This work
pET-S-2-G	S-2-G gene on pET-28a, 7.66 kb	This work
pET-G-S-2	G-S-2 gene on pET-28a, 7.66 kb	This work
pET-G-2-S	G-2-S gene on pET-28a, 7.66 kb	This work

Primers	Sequence (5′→3′)	
SCRII-F_1_	GGATCCATGGGCGAAATCGAATCTTATTGCAA	
SCRII-F_2_	TGGCCGCGGT**GAAGGAGATATACC**ATGGGCGAAATCGAATCTTA	
SCRII-F_3_	TACCGTGAGC**GAAGGAGATATACC**ATGGGCGAAATCGAATCTTA	
SCRII-R_1_	AACTCACCAT**GGTATATCTCCTTC**TGGACAAGTGTAACCACCATCG	
SCRII-R_2_	CCGGATACAT**GGTATATCTCCTTC**TGGACAAGTGTAACCACCATCG	
SCRII-R_3_	TGGTCTGCAT**GGTATATCTCCTTC**TGGACAAGTGTAACCACCATCG	
SCRII-R_4_	GAGCTCTGGACAAGTGTAACCACCATCG	
A258F/GDH-F_1_	TACCGTGAGC**GAAGGAGATATACC**ATGTATCCGGATTTAAAAGG	
A258F/GDH-F_2_	CACTTGTCCA**GAAGGAGATATACC**ATGTATCCGGATTTAAAAGG	
A258F/GDH-F_3_	GGATCCATGTATCCGGATTTAAAAGG	
A258F/GDH-R_1_	GAGCTCACCGCGGCCAAACTGGAATG	
A258F/GDH-R_2_	TGGTCTGCAT**GGTATATCTCCTTC**ACCGCGGCCAAACTGGAATG	
A258F/GDH-R_3_	TTTCGCCCAT**GGTATATCTCCTTC**ACCGCGGCCAAACTGGAATG	
XYN2-F_1_	CACTTGTCCA**GAAGGAGATATACC**ATGCAGACCATCCAGCCGGG	
XYN2-F_2_	TGGCCGCGGT**GAAGGAGATATACC**ATGCAGACCATCCAGCCGGG	
XYN2-F_3_	CACTTGTCCA**GAAGGAGATATACC**ATGCAGACCATCCAGCCGGG	
XYN2-R_1_	CCGGATACAT**GGTATATCTCCTTC**GCTCACGGTAATGCTGGCGC	
XYN2-R_2_	TTTCGCCCAT**GGTATATCTCCTTC**GCTCACGGTAATGCTGGCGC	
XYN2-R_3_	GAGCTCGCTCACGGTAATGCTGGCGC	

The sequence of SD-AS is bold; the restriction endonuclease sites are underlined

*Ap*^*r*^ ampicillin resistance

### Gene cloning of SCRII, A258F/GDH and XYN2

The oligonucleotide primers (Table 2) were designed based on the gene sequences. The genes SCRII, A258F/GDH and XYN2 were amplified using the plasmids pET-SCRII, pET-A258F/GDH and pET-XYN2 as the DNA template, respectively. The PCR-amplified products were ligated to pMD19-T (Takara-Bio, Kyoto, Japan) to obtain T-SCRII, T-A258F/GDH and T-XYN2 plasmids, which were transformed in *E. coli* JM109 cells and the recombinant strains were verified by DNA sequencing in Takara Co. (Shanghai, China).

### Construction of co-expression system of SCRII, A258F/GDH and XYN2

Several multi-enzymes coupled systems containing the SCRII, XYN2 and A258F/GDH were constructed using a Shine–Dalgarno (SD) and aligned spacing (AS) sequence (GAAGGAGATATACC) linker between them. Either SCRII or A258F/GDH was nearest to the promoter. The fusion genes SCRII-SD-AS-A258F/GDH-SD-AS-XYN2 (named as S-G-2), SCRII-SD-AS-XYN2-SD-AS-A258F/GDH (named as S-2-G), A258F/GDH-SD-AS-SCRII-SD-AS-XYN2 (named as G-S-2), A258F/GDH-SD-AS-XYN2-SD-AS-SCRII (named as G-2-S), were cloned using overlap-extension technique. In each fusion gene, the leftmost genes were nearest the promoter. Then the four fusion genes were constructed on the plasmid pET-28a, and the plasmids, pET-S-G-2, pET-S-2-G, pET-G-S-2 and pET-G-2-S were transformed into the competent cells of *E. coli* BL21. The corresponding recombinant strains *E. coli*/pET-S-G-2, *E. coli*pET-S-2-G, *E. coli*pET-G-S-2 and *E. coli*pET-G-2-S were obtained after the confirmation of nucleotide sequencing. Meanwhile, the plasmids, pET-SCRII, pET-A258F/GDH, and pET-XYN2 were transformed into the competent cells of *E. coli* BL21 to obtain recombinant *E. coli*/pET-SCRII, *E. coli*/pET-A258F/GDH and *E. coli*/pET-XYN2.

### Protein expression

The recombinant strains *E. coli*/pET-S-2-G, *E. coli*/pET-S-G-2, *E. coli*/pET-G-2-S and *E. coli*/pET-G-S-2 were cultured in LB medium containing 50 μg/mL kanamycin at 37 °C. When *OD*_600_ value of the culture reached 0.8, isopropyl-β-D-thiogalactopyranoside (IPTG) of 0.1 mM was added to induce protein expression. The cultures were cultivated at 25 °C for 16 h. The cultures were harvested by centrifugation, suspended in 50 mM Tris–HCl (pH 8.0) and 150 mM NaCl, and then disrupted with an ultrasonic oscillator (Insonater 201 M; Kubota, Japan). After centrifugation (10,000×*g*) for 30 min at 4 °C, the cell-free extracts were used for sodium dodecyl sulfate–polyacrylamide gel electrophoresis (SDS-PAGE) analysis and the enzyme assays.

### Enzyme assay

The specific activities of three enzymes, SCRII, A258F/GDH and XYN2 were determined using cell-free extracts of recombinant *E. coli*/pET-G-S-2, *E. coli*/pET-G-2-S, *E. coli*/pET-S-G-2 and *E. coli*/pET-S-2-G. Their enzyme activities were analyzed using 2-HAP, xylose and xylan as substrates, respectively.

The enzymatic activities of SCRII for oxidation of 2-HAP were measured at 35 °C and pH 6.5 mixture by spectrophotometrically recording the rate of change of NADPH absorbance at 340 nm. One unit of enzyme activity is defined as the amount of enzyme catalyzing the oxidation of 1 μmol of NADPH per minute under the measurement conditions. The standard assays were performed as described by Zhang et al. [23].

The oxidation activities of A258F/GDH were measured at 35 °C and pH 7.0 by spectrophotometrically recording the rate of change of NADPH absorbance at 340 nm. One unit of enzyme activity is defined as the amount of enzyme catalyzing the reduction of 1 μmol of NADP^+^ per minute under the measurement conditions.

Xylanase activity was assayed by the method described by Bailey et al. with 1% oat-spelt xylan (Sigma) as substrate at 50 °C [31]. Appropriate dilutions of the protein solution in 0.1 M sodium citrate buffer (pH 6.0) were used as the enzyme source. The amount of released sugar was determined by the dinitrosalicylic acid method described by Miller et al. [32]. The protein concentration was determined by the Bradford method with bovine serum albumin as the standard.

The temperature optimum of enzymes activity was determined at various temperatures (20–80 °C). The pH optimum of enzyme activity was determined at the optimal temperature over a pH range of 3.0–10.0. The buffers used were 0.1 M sodium citrate buffer (pH 3.0 to 6.5), 0.1 M potassium phosphate buffer (pH 6.5 to 7.5), and 0.1 M Tris–HCl buffer (pH 8.0 to 10.0), respectively.

### Biotransformation and analytical methods

The recombinant *E. coli*/pET-G-S-2 cells were used for 2-HAP biotransformation. The biotransformation was carried out as described previously with minor modifications [33]. The reaction mixture (2 mL) consisted of 0.1 M sodium citrate buffer (pH 6.5), 6 g/L 2-HAP, 6 g/L xylan, and 0.2 g washed wet cells (10% w/v). When the reaction was upscaled in 200 mL, the corresponding washed wet cells was 20 g, and the other components in the reaction mixture maintained the same concentration. The reactions were carried out at 35 °C for 24 h with shaking at 200 rpm, using the wet recombinant cells as biocatalysts. At the end of the reaction, the product (*S*)-PED was extracted with ethyl acetate, and the organic layer was used for analysis. The optical purity and yield of the product were determined by HPLC on a Chiralcel OB-H column (4.6 × 250 mm Daicel Chemical Ind. Ltd., Japan) with flow rate 0.5 mL/min at 25 °C. The retention times of (*S*)-PED and 2-HAP are 11.5 and 17.8 min, respectively.

### pH and temperature dependence

The effects of pH and reaction temperature on 2-HAP biotransformation were determined by the whole cells of *E. coli*/pET-G-S-2. The biotransformation of 2-HAP to (*S*)-PED was carried out in pH 3.0–10.0 using 0.1 M sodium citrate (pH 3.0–6.5), 0.1 M potassium phosphate (pH 6.5–7.5), and 0.1 mM Tris–HCl (pH 8.0–10.0) as buffer. The temperature dependence of *E. coli*/pET-G-S-2 mediated 2-HAP transformation was determined at various temperatures ranging from 20 to 50 °C under the optimal pH. The biotransformation of 2-HAP was determined with the standard assay method described above.

### Optimization of ratios of substrate and co-substrate

Under the optimal pH and temperature, the biotransformation was explored by *E. coli/*pET-G-S-2 with the ratios of 2-HAP and xylan varying from 5:1 to 1:5. The effects of the ratios on the efficiency of 2-HAP transformation were configured with 6 g/L 2-HAP and 1.2, 2.0, 3.0, 6.0, 12.0, 18.0 or 30.0 g/L xylan.

## Data Availability

The datasets of supporting the conclusions in this article are included in the manuscript.

## Abbreviations

GDH: Glucose dehydrogenase
2-HAP: 2-Hydroxyacetophenone
SCRII: (*S*)-carbonyl reductase II
(*S*)-PED: (*S*)-1-phenyl-1, 2-ethanediol
XYN2: Endo-β-1,4-xylanase 2
